# Spectrum of cardiovascular diseases at a referral tertiary care hospital in Somalia, Mogadishu: an echocardiographic study

**DOI:** 10.1186/s12872-021-02417-4

**Published:** 2021-12-16

**Authors:** Gökhan Alıcı, Ömer Genç

**Affiliations:** 1 Turkey, Recep Tayyip Erdogan, Somalia Mogadishu Training and Research Hospital, Mogadishu, Somalia; 2 Department of Cardiology, Agri Training and Research Hospital, Agri, Turkey

**Keywords:** Congenital heart diseases, Rheumatic heart disease, Somalia, Hypertensive heart disease, Valvular heart disease

## Abstract

**Background:**

To investigate the frequencies and patterns of cardiovascular diseases (CVDs), including rheumatic and congenital heart diseases, among patients with abnormal hearts assessed by echocardiographic examination.

**Methods:**

This retrospective, descriptive registry reviewed abnormal echocardiographic findings of 1140 patients aged 0–100 years who were admitted to the cardiology outpatient clinic at a tertiary training institution in Mogadishu.

**Results:**

Hypertensive heart disease (HHD) (n:454, 39.8%), valvular heart disease (VHD) (n:395, 34.6%), and heart failure with reduced ejection fraction (HFrEF) (n:351, 30.8%) were the most frequent comorbidities. Congenital heart diseases (CHDs) were detected in 151 (13.2%) of the patients, with the most common ones including atrial septal defect (ASD) (n:37, 3.2%) and ventricular septal defect (VSD) (n:26, 2.3%). Rheumatic heart disease (RHD) was observed in 84 (7.4%) patients, among whom the most common age range was 16–30 years (40.5%), followed by 31–45 years (31%) and 0–15 years (15.5%). Mitral insufficiency (n:541, 47.5%) was detected as the most frequent VHD, followed by aortic insufficiency (n:437, 38.3%), and tricuspid insufficiency (n:264, 23.2%) and mitral valve stenosis (n:39, 3.4%) was the least common VHD.

**Conclusion:**

In the present study, we found that HHD was the most common comorbidity, followed by VHD, and HFrEF. Moreover, the most common VHD was mitral insufficiency and the most common CHD was ASD.

**Supplementary Information:**

The online version contains supplementary material available at 10.1186/s12872-021-02417-4.

## Introduction

Cardiovascular diseases (CVDs) are the leading cause of death worldwide [1]. The frequency, treatment options, subgroups including congenital heart diseases (CHDs) and rheumatic heart disease (RHD), and outcomes have been well documented in developed countries so far. There are, however, still serious concerns regarding the quality and reliability of available data in low-middle income countries. According to the World Health Organization, CVDs are the second most common cause of overall mortality in Africa. Further supporting this conclusion, 1.2 million people worldwide died from CVDs in 2015 [2]. Of note, CHDs was significantly related to poor cardiovascular outcome among young children and adolescents living in Africa [3]. Meanwhile, difficulties caused by deficiencies in genetic testing and/or advanced imaging methods may cause the diagnosis to be missed or delayed. However, unlike CHDs, which have a relatively similar distribution all over the world, RHD resulting from damage to the heart valves caused by one or more episodes of rheumatic fever is naturally preventable and also another important contributor to morbidity and mortality in low-middle income countries [4]. Furthermore, RHD remains a devastating impact on the health system and is associated with approximately 300,000 deaths globally and loss of > 10 million disability-adjusted life years [5].

Even though echocardiography is an easy-to-use, cost-effective, non-invasive, and reliable ultrasound-based modality, its use in many parts of Africa is still highly limited [6]. Therefore, there is a lack of reliable data on diagnosis, follow-up, treatment, and prevention of CVDs in those regions. Moreover, this drawback is considered to be a crucial and noteworthy challenge in reducing preventable non-communicable diseases in low-middle-income countries. To this end, the present study sought to address the frequency and pattern of CVDs assessed by echocardiographic examinations in Mogadishu, the capital of Somalia.

## Patients and methods

### Study population and design

**Fig. 1:**
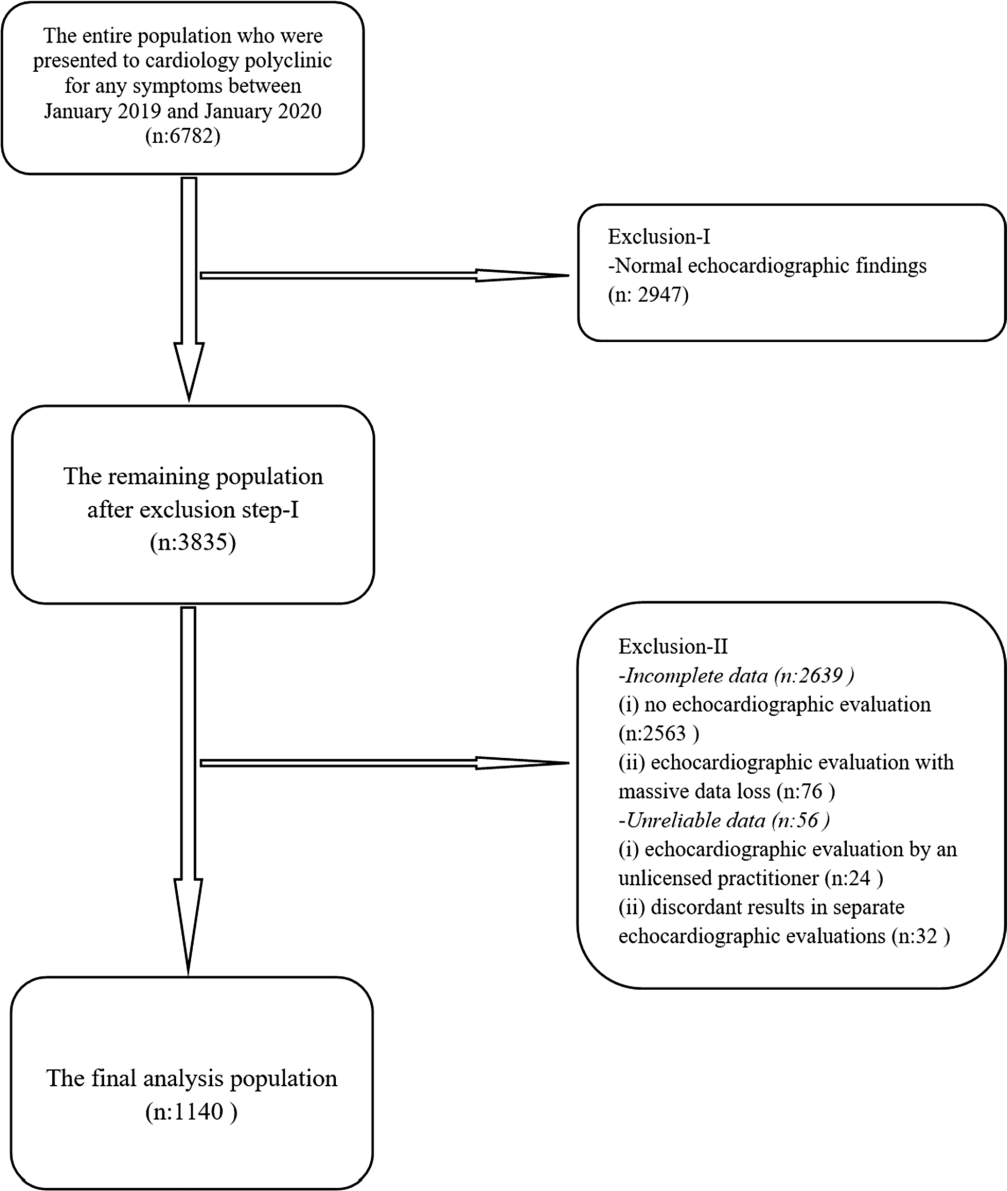
Flowchart of inclusion/exclusion process in the study

This retrospective, descriptive and observational registry reviewed the echocardiographic findings of patients aged 0–100 years who were presented to our outpatient cardiology clinic at a tertiary training hospital in Mogadishu, between January 1, 2019, and January 1, 2020. Overall, 6782 subjects admitted to the hospital were screened. 5642 individuals who had incomplete, unreliable data and/or those with completely normal echocardiographic findings were excluded from the analysis (Fig. 1). Accordingly, a total of 1140 patients with abnormal echocardiographic findings by age group and gender were enrolled in the study. Demographic characteristics and echocardiographic parameters including left ventricular ejection fraction (LVEF), interventricular septum thickness, left ventricular (LV) diastolic dysfunction grade, mitral valve insufficiency/stenosis, and rheumatic, and congenital heart diseases were analyzed for each participant. Echocardiographic evaluations were performed by experienced echocardiographers who were licensed in Turkey using a Toshiba Aplio™ ultrasound system (TUS-A500, Shimoishigami, Japan) in accordance with the American Society of Echocardiography guidelines [7]. Those aged 15 and under were defined as children. Age- and gender-based distributions of acquired, congenital, and rheumatic heart diseases were reported. Those with tuberculosis (active or previous) were evaluated together, regardless of whether they received treatment or not. The study was conducted according to the Helsinki Declaration. Ethical approval was obtained from the local ethics committee (date: 17.02.2021, decision no: 323). The need for informed consent was waived due to the retrospective nature of the study.

Normal echocardiography was defined as echocardiographic evaluation in which bi-ventricle (both in size and function) and valves are within normal ranges by gender and age group [8, 9]**.**

Hypertensive heart disease (HHD) was diagnosed in the presence of signs of heart failure or criteria for concentric/eccentric LV hypertrophy (wall diameters, cavity size, and left ventricular mass index in echocardiography [10] or diagnostic electrocardiography (ECG) parameters [11]), left atrial enlargement (anteroposterior diameter, left atrial volume index measured by echocardiography or left atrial dilatation criteria on ECG), and/or presence of at least one of the following parameters; (a) severe left ventricular diastolic dysfunction (E/e’ > 14), (b) left ventricular systolic dysfunction (LVEF < 50%), (c) systolic pulmonary arterial hypertension > 60 mmHg, (d) valvular insufficiency (mitral or tricuspid) considered not to be caused by valvular heart disease (VHD) and/or acute ischemic heart disease, for known or newly diagnosed hypertensive patients (blood pressure > 140/90 mmHg).

Valvular heart disease (VHD) was defined as an obvious function and size abnormality with the calculation of velocity (m/s), gradient (mmHg), and area (cm^2^) for stenotic valves and with the interpretation of qualitative (valve morphology, colour flow, and holodiastolic flow reversal in descending aorta), semiquantitative (vena contracta width, pressure half time), and quantitative (regurgitant volume, effective regurgitant orifice area) measurements for valve insufficiency in at least one of the heart valves according to European Society of Cardiology guidelines for the management of valvular heart disease [12].

Rheumatic heart disease (RHD) was diagnosed in accordance with the 2012 World Heart Federation criteria for echocardiographic diagnosis of RHD [13]. Criteria include pathological (seen in two views, jet length, velocity, pan-systolic/pan-diastolic jet in at least one envelope) and morphological features (thickening, restricted leaflet motion, prolapse, coaptation defect, excessive leaflet tip motion) for valve regurgitation, and a gradient increase of ≥ 4 mmHg in mitral stenosis.

Ischemic heart disease (IHD) was diagnosed in patients with angina pectoris (current or past), previous myocardial infarction, and/or documented coronary artery disease, or an ECG feature indicating a previous myocardial infarction and/or a regional wall motion abnormality suggestive of myocardial infarction detected in echocardiography.

Dilated cardiomyopathy (DCMP); LV or biventricular systolic dysfunction and dilatation that are not explained by abnormal filling conditions, regardless of being primary or secondary. Systolic dysfunction is defined by abnormal LVEF < 45%, and LV dilatation is defined by LV end-diastolic diameters > 2 standard deviations from normal according to normograms corrected by body surface area and age [14].

Hypertrophic cardiomyopathy (HCMP) was defined as unexplained maximal wall thickness > 15 mm in any LV myocardial segment or presence of LV septal/posterior wall thickness ratio > 1.3 in normotensive patients and > 1.5 in hypertensive patients [15, 16]. Since HCMP patients in our study were adults, both thresholds were applied to all of them.

Pericardial effusion was diagnosed in the presence of an echo-free space between the visceral and the parietal pericardium. The classification was as follows: mild (< 10 mm), moderate (10-20 mm), and severe (> 20 mm).

Constrictive pericarditis was diagnosed with the help of conventional imaging methods (chest X-ray and computerized tomography-thickened, calcified, fibrotic pericardium-) and certain echocardiographic findings (including respirophasic ventricular septal shift-septal bounce-, hepatic vein diastolic flow reversal with expiration, preserved/exaggerated medial mitral annulus early diastolic (e′) velocity of ≥ 9 cm/s, at least > 25% respiratory variation of peak mitral E-wave velocity, and medial e′/lateral e′ ≥ 0.91), together with the symptoms and signs of heart failure.

Heart failure with reduced EF (HFrEF) was diagnosed in the presence of risk factors, abnormal ECG, clinical signs (e.g. elevated jugular venous pressure, hepatojugular reflux, laterally displaced apical impulse), and/or symptoms (e.g. reduced exercise tolerance, paroxysmal nocturnal dyspnea, breathlessness, orthopnoea) of heart failure, along with a reduced EF of < 50% assessed by echocardiography. Subjects with an LVEF between 41 and 49% were defined as mildly reduced LV systolic dysfunction [17].

Diastolic dysfunction, which refers to impaired LV relaxation, with or without an increase in filling pressure, was categorized into 4 grades. Grade I (mild diastolic dysfunction: E/A < 0.8, deceleration time (DT) > 200 ms, average E/e′ ≤ 8), grade II (moderate diastolic dysfunction or pseudonormal phase: E/A 0.8–1.5, DT 160–200 ms, average E/e′ 9–12), grade III (severe diastolic dysfunction or reversible restrictive filling phase: E/A ≥ 2, DT < 160 ms, average E/e′ ≥ 13), and grade IV (irreversible/fixed restrictive filling phase: as grade III with no benefit from a reduction of preload) [18].

Pulmonary arterial hypertension was defined as the presence of systolic pulmonary artery pressure (SPAP) ≥ 2.8 m/sec or ≥ 36 mmHg in echocardiography, in addition to symptoms and other findings that are associated with pulmonary hypertension [19].

### Statistical analysis

Statistical analyses were performed using IBM SPSS Statistics for Windows Version 20.0 (Armonk, NY: IBM Corp.). The normality of continuous variables was assessed by analytical (Kolmogorov–Smirnov test) and visual methods (histograms and probability plots). All continuous variables were expressed as median (interquartile range [IQR]) due to the presence of abnormal distribution. Categorical variables were expressed as number (n) and percentage (%). Continuous variables were compared using the Mann–Whitney U test and categorical variables were compared using the χ^2^-test or Fisher’s exact test, where appropriate. A two-tailed *p* value of < 0.05 was considered significant throughout the study.

## Results

**Fig. 2:**
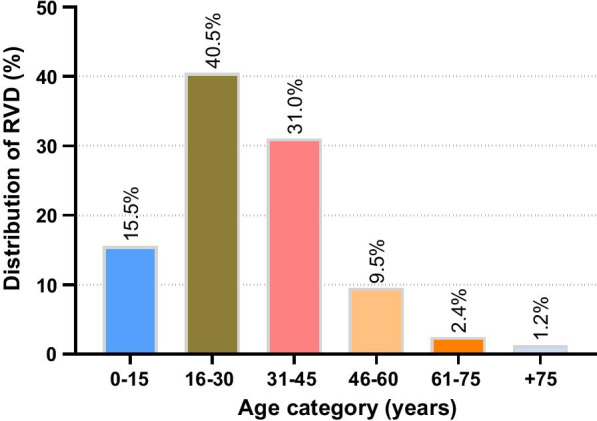
Distribution of disorder by age category among patients with rheumatic valvular disease. *RVD* rheumatic valvular disease

Out of the study population, 646 (56.7%) were male, the median age was 60 (IQR; 42–70) years and 113 (9.9%) were children. Children were more likely to be female (*p* = 0.045). Overall, HHD was found to be the most common comorbidity (n:454, 39.8%), followed by VHD (n:395, 34.6%), HFrEF (n:351, 30.8%), and IHD (n:278, 24.4%). RHD was detected in 84 (7.4%) patients. Males tended to have older age, higher rates of diabetes mellitus, HHD, IHD, HFrEF, and DCM, whereas females had more frequent RHD (*p* < 0.05, for all). While the number of patients with tuberculosis was 202 (17.7%), constrictive pericarditis was diagnosed for various reasons in 9 (0.8%). Detailed demographic characteristics of the study population are listed in Table 1. In subgroup analysis, CHDs were statistically more common in children than in adults (n:102, 90.3% vs n:49, 4.8%, *p* < 0.001). On the other hand, HHD, IHD, HFrEF, VHD, and DCM were detected at higher rates in adults (*p* < 0.05, for all) (Additional file 1). The most observed age range among patients with RHD was 16–30 years (n:34, 40.5%), followed by 31–45 years (n:26, 31%), and 0–15 years (n:13, 15.5%) (Fig. 2).

**Table 1:** Demographic characteristics of the study population by gender

Variables	All (n:1140)	Male (n:646)	Female (n:494)	*p* value
Age, years	60 (42–70)	61 (47–71)	60 (35–70)	**0.002**
Child, n (%)	113 (9.9)	54 (8.4)	59 (11.9)	**0.045**
Diabetes mellitus, n (%)	263 (23.1)	183 (28.3)	80 (16.2)	< **0.001**
Hypertensive heart disease, n (%)	454 (39.8)	274 (42.4)	180 (36.4)	**0.041**
Current smoker, n (%)	57 (5.0)	26 (4.0)	31 (6.3)	0.084
Ischemic heart disease, n (%)	278 (24.4)	206 (31.9)	72 (14.5)	< **0.001**
Heart failure with reduced EF, n (%)	351 (30.8)	237 (36.7)	114 (23.1)	< **0.001**
Mildly reduced (41–49%)	59 (5.2)	33 (5.1)	26 (5.3)	
Reduced (≤ 40%)	292 (25.6)	204 (31.6)	88 (17.8)	
Valvular heart disease, n (%)	395 (34.6)	234 (36.2)	161 (32.6)	0.555
Mild	170 (14.9)	104 (16.1)	66 (13.4)	
Moderate	166 (14.6)	96 (14.9)	70 (14.2)	
Severe	59 (5.2)	34 (5.3)	25 (5.1)	
Rheumatic heart disease, n (%)	84 (7.4)	30 (4.6)	54 (10.9)	< **0.001**
Congenital heart disease, n (%)	151 (13.2)	75 (11.6)	76 (15.4)	0.062
Pulmonary arterial hypertension, n (%)	260 (22.8)	150 (23.2)	110 (22.3)	0.745
COPD, n (%)	66 (5.8)	13 (2.0)	53 (10.7)	< **0.001**
Dilated cardiomyopathy, n (%)	225 (19.7)	160 (24.8)	65 (13.2)	< **0.001**
Constrictive pericarditis, n (%)	9 (0.8)	7 (1.1)	2 (0.4)	0.173
Peripartum cardiomyopathy, n (%)	21 (1.8)	–	21 (4.3)	–
Tuberculosis, n (%)	202 (17.7)	104 (16.1)	98 (19.8)	0.101

Bold indicates *p* value < 0.05 was considered significant

Data are presented as number (n) and percentage (%), or median (interquartile range). *p* value was calculated using the Mann–Whitney U-test for continuous variables and the Chi-Square test or the Fisher's exact test for categorical variables as appropriate. *p* value < 0.05 was considered significant. *COPD* chronic obstructive pulmonary disease, *EF* ejection fraction

**Fig. 3:**
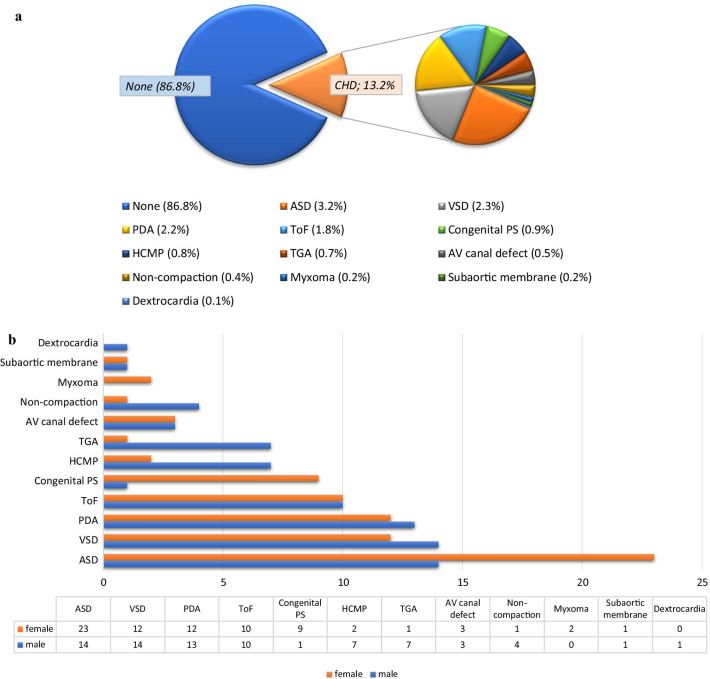
**a** Distribution of various patterns of congenital heart diseases. **b** Numerical distribution chart of congenital heart diseases by gender. *PDA* Patent ductus arteriosus, *HCMP* hypertrophic cardiomyopathy, *ASD* atrial septal defect, *VSD* ventricular septal defect, *ToF* tetralogy of Fallot, *TGA* transposition of the great arteries, *Congenital PS* Congenital pulmonary stenosis, *AV canal defect* atrioventricular canal defect, *CHD* congenital heart disease

Median EF in the entire population was 60% (IQR: 40–65) and lower in males than in females [55 (35–60) vs 60 (55–65), *p* < 0.001]. There were 182 (15.9%) subjects with grade II-IV diastolic dysfunction. Overall, 59 (5.2%) subjects had severe valvular pathology (regurgitation/stenosis) in at least one valve, while 336 (29.5%) had mild to moderate valve pathology in at least one valve. Mitral insufficiency (n:541, 47.5%) was detected as the most frequent VHD, followed by aortic insufficiency (n:437, 38.3%), and tricuspid insufficiency (n:264, 23.2%) and mitral valve stenosis (n:39, 3.4%) was the least common VHD (Table 2). On the other hand, 151 (13.2%) patients had CHDs, of whom 23 (15.2%) were cyanotic CHDs. The most frequent CHD was atrial septal defect (ASD) (n:37, 3.2%), followed by ventricular septal defect (VSD) (n:26, 2.3%) and patent ductus arteriosus (PDA) (n:25, 2.2%) (Fig. 3a). ASD was observed to be more common in females, whereas VSD and PDA had a similar frequency across genders (Fig. 3b). When we analyzed the age distribution of participants with CHDs, the most frequent age group was 0–15 years (67.5%), followed by 16–30 years (19.2%) (Table 3).

**Table 2:** Echocardiographic findings of the study population by gender

Variables	All (n:1140)	Male (n:646)	Female (n:494)	*p* value
Ejection fraction, %	60 (40–65)	55 (35–60)	60 (55–65)	< **0.001**
Interventricular septum thickness, mm	11 (10–14)	13 (10–14)	11 (10–14)	**0.001**
LV diastolic dysfunction and grades, n (%)	668 (58.6)	416 (64.4)	252 (51.0)	< **0.001**
Grade I	486 (42.6)	289 (44.7)	197 (39.9)	
Grade II	141 (12.4)	99 (15.3)	42 (8.5)	
Grade III	34 (2.9)	23 (3.6)	11 (2.2)	
Grade IV	7 (0.6)	5 (0.8)	2 (0.4)	
Aortic valve insufficiency, n (%)	437 (38.3)	260 (40.2)	177 (35.8)	0.233
Mild	340 (29.8)	209 (32.4)	131 (26.5)	
Moderate	81 (7.1)	42 (6.5)	39 (7.9)	
Severe	16 (1.4)	9 (1.4)	7 (1.4)	
Aortic valve stenosis, n (%)	87 (7.6)			0.118
Mild	52 (4.6)	25 (3.9)	27 (5.5)	
Moderate	9 (0.8)	5 (0.8)	4 (0.8)	
Severe	26 (2.3)	20 (3.1)	6 (1.2)	
Mitral valve insufficiency, n (%)	541 (47.5)	322 (49.8)	219 (44.3)	0.135
Mild	263 (23.1)	150 (23.2)	113 (22.9)	
Moderate	254 (22.3)	154 (23.8)	100 (20.2)	
Severe	24 (2.1)	18 (2.8)	6 (1.2)	
Mitral valve stenosis, n (%)	39 (3.4)	13 (2.0)	26 (5.3)	**0.003**
Mild	18 (1.6)	9 (1.4)	9 (1.8)	
Moderate	8 (0.7)	1 (0.2)	7 (1.4)	
Severe	13 (1.1)	3 (0.5)	10 (2.0)	
Tricuspid valve insufficiency, n (%)	264 (23.2)	153 (23.7)	111 (22.5)	0.880
Systolic pulmonary artery pressure, mmHg	20 (15–30)	22 (15–30)	18 (20–30)	0.776
Valvular vegetation, n (%)	2 (0.2)	1 (0.2)	1 (0.2)	0.677
Left ventricular thrombus, n (%)	5 (0.4)	5 (0.8)	0 (0)	0.074
Permeant pacemaker lead, n (%)	6 (0.5)	5 (0.8)	1 (0.2)	0.244
Pericardial effusion, n (%)	125 (10.9)	70 (10.8)	55 (11.1)	0.326
Mild	114 (10.0)	65 (10.1)	49 (9.9)	
Moderate	8 (0.7)	4 (0.6)	4 (0.8)	
Severe	3 (0.3)	1 (0.2)	2 (0.4)	

Bold indicates *p* value < 0.05 was considered significant

Data are presented as numbers and percentages (%), or median (interquartile range). p-value was calculated using the Mann–Whitney U-test for continuous variables and the Chi-Square test or the Fisher's exact test for categorical variables as appropriate. p value < 0.05 was considered significant

**Table 3:** Age-based distribution of congenital heart diseases

Age category, years	Non-compaction n (%)	ASD n (%)	AVCD n (%)	PDA n (%)	Congenital PS n (%)	Dextrocardia n (%)	ToF n (%)	HCMP n (%)	Myxoma n (%)	TGA n (%)	VSD n (%)	Subaortic membrane n (%)	Percentage among CHDs, n (%)
0–15	0 (0)	25 (2.2)	5 (0.4)	23 (2.0)	7 (0.6)	0 (0)	15 (1.3)	0 (0)	0 (0)	8 (0.7)	18 (1.6)	1 (0.1)	102 (67.5)
16–30	1 (0.1)	8 (0.7)	1 (0.1)	2 (0.2)	3 (0.3)	0 (0)	4 (0.4)	4 (0.4)	0 (0)	0 (0)	5 (0.4)	1 (0.1)	29 (19.2)
31–45	0 (0)	1 (0.1)	0 (0)	0 (0)	0 (0)	0 (0)	1 (0.1)	1 (0.1)	1 (0.1)	0 (0)	2 (0.2)	0 (0)	6 (4.0)
46–60	3 (0.3)	2 (0.2)	0 (0)	0 (0)	0 (0)	1 (0.1)	0 (0)	4 (0.4)	1 (0.1)	0 (0)	1 (0.1)	0 (0)	12 (7.9)
61–75	1 (0.1)	1 (0.1)	0 (0)	0 (0)	0 (0)	0 (0)	0 (0)	0 (0)	0 (0)	0 (0)	0 (0)	0 (0)	2 (1.3)
+ 75	0 (0)	0 (0)	0 (0)	0 (0)	0 (0)	0 (0)	0 (0)	0 (0)	0 (0)	0 (0)	0 (0)	0 (0)	0 (0)
Percentage among the study population	5 (0.4)	37 (3.2)	6 (0.5)	25 (2.2)	10 (0.9)	1 (0.1)	20 (1.8)	9 (0.8)	2 (0.2)	8 (0.7)	26 (2.3)	2 (0.2)	

*ASD* atrial septal defect, *AVCD* atrioventricular canal defect, *PDA* patent ductus arteriosus, *PS* pulmonary stenosis, *ToF* tetralogy of Fallot, *HCMP* hypertrophic cardiomyopathy, *TGA* transposition of the Great Arteries, *VSD* ventricular septal defect

## Discussion

To the best of our knowledge, this is the first echocardiographic study to comprehensively evaluate such a large cohort of patients in Somalia. Given inadequate healthcare services in Somalia, which is an active war zone, we consider that this study is of high value. In our investigation, HHD was the most frequent comorbidity (39.8%), followed by VHD (34.6%), HFrEF (30.8%), and IHD (24.4%), RHD (7.4%). Additionally, 13.2%, the majority of whom were children, of the study population were diagnosed with CHDs, with the most common being ASD, VSD, and PDA, respectively.

Somalia's total population is estimated at 15.4 million, of whom about 4.5 million are children under the age of 14. Health institutions in Somalia often provide rather limited facilities for individuals with chronic illnesses and those in need of advanced care and unfortunately, no tertiary healthcare centers exist in Mogadishu and surrounding provinces, except for the region where this study was conducted. All these deficiencies prevent timely diagnosis by appropriate detection modalities, increasing the likelihood of poor outcomes in such populations. Moreover, delayed hospital admission, advanced maternal age, high rates of consanguineous marriage, and the presence of many infectious diseases that have not been eradicated nationwide may result in an increased incidence and prevalence of both acquired and congenital heart diseases. Yet, unfortunately, there are very few studies in the literature reporting the risk factors, profile, and echocardiographic characteristics of the African population. For instance, in an echocardiographic study conducted in Nigeria, Ogah et al. [20] determined HHD in 56.7% of the patients. In another echocardiographic study, Raphael et al. [21] evaluated 815 adults and 59 children aged ≤ 15 years and reported that normal echocardiographic findings were detected in only 44% of the patients and that the most common comorbidities were HHD (41%), VHD (18%), coronary artery disease (18%), and peripartum cardiomyopathy (7%) in adults, while CHDs in children (34%). In our study, HHD was also the most common co-morbidity (39.8%), which could be related to the excessive salt consumption by habitants in the region and also to some other factors such as difficult and delayed diagnosis, inadequate medication, and loss to follow-up.

Despite the decrease in the incidence of rheumatic fever/RHD over the last few decades, it remains one of the leading acquired health problems, especially in low-middle income countries [22]. In an investigation on patients with structural and functional valve abnormalities, 24% of the subjects had a valvular abnormality, with the most frequent abnormality being mitral valve insufficiency (59%), and approximately one-third (36%) of the patients were diagnosed with RHD [23]. In the present study, the most frequent valvular abnormality was mitral insufficiency (47.5%), consistent with the former, and a high rate of RHD was diagnosed. Additionally, the relationship between severe VHD and mortality has been demonstrated [24, 25], regardless of the cause, and it is very important to determine the severity of VHD in terms of both follow-up and treatment options. Precisely in this regard, despite not having follow-up records, we have revealed severe valve pathology (insufficiency/stenosis) in 5.2% of the study population with abnormal echocardiographic findings, which is significant data that should be addressed in order to reduce the poor outcome in the region. As noted above, RHD is a very important health problem that needs to be thoroughly addressed in many African countries, including Somalia, mostly due to inadequate healthcare. Also, it is an inevitable requirement to improve healthcare systems and patient follow-up to reduce the incidence of RHD associated with autoimmune pathophysiology.

The prevalence of CHDs is relatively similar worldwide, ranging from 4 to 85 per 1000 births [4, 26, 27]. Although VSD is generally the most common CHDs, studies with a higher frequency of ASD have also been reported, which is compatible with ours [28, 29]. This discordance may be related to the following conditions; (1) inter-regional genetic variation, (2) the overwhelming majority of the participants in our study population is adults and we acknowledge that some small VSDs might close spontaneously with age, and (3) diagnosis by transthoracic echocardiography alone may cause some VSDs to be overlooked. Besides, factors such as excessive use of herbal medicines, alcohol-cigarette consumption, inadequate control of chronic diseases, and the insufficient number of vaccination doses against infectious diseases may be associated with the high prevalence of CHDs in Somalia [30]. Nowadays, with the support of advances in technology and industry, the prevalence of CHDs in adulthood, also called adult congenital heart diseases, has increased steadily in developed countries, whereas in countries with limited resources such as Somalia, only a small proportion of patients with CHDs, particularly in the “complex” subgroup, can reach adulthood, mainly due to deaths from major adverse cardiovascular events and related infections. Similarly, in our study, we concluded that 67.5% of individuals with CHDs were 15 years of age or younger.

## Clinical implications

As might be predicted, our study intends to raise public awareness rather than introduce innovation or examine a relationship. In this sense, the study will partially fill the data gap in Somalia, which is a war zone. Also, the presence of high rates of HHD, CHDs, and VHD reported in the study sheds light on the points to be improved in the healthcare system so that limited opportunities could be directed to the right areas. Most importantly, the serious lack of accurate diagnosis, treatment, follow-up, and data recording stages in the design and implementation phase have made the current study very difficult and may adversely affect the continuity and planning of other researches in the future. Therefore, we find it clinically crucial to articulate this challenge.

### Limitations and strengths

#### Limitations

First, a single-center and retrospective design of the study may be the main limitation for the generalizability of the study. Second, only patients with abnormal findings detected by echocardiography were included in the study and thus the incidence and prevalence of the diseases and risk factors could not be evaluated. Third, there could be errors in data collection, diversification, and standardization due to the absence or lack of diagnostic techniques or confirmation processes including transesophageal echocardiography, coronary angiography, cardiac catheterization, telephone interview, and address records. Fourth, the classification of CVDs may overlap due to the study design being only echo-based. It is likely that several patients with VHD also have RHD. Fifth, CHDs types such as bicuspid aortic valve, aortic coarctation, truncus arteriosus, single ventricle, etc. could not be diagnosed, probably due to the lack of diagnostic instruments and healthcare services mentioned above. Therefore, the distribution of CHDs in the general population is not fully reflected. Sixth, due to the retrospective nature of the study and country-specific conditions, echocardiographic evaluation was made by different echocardiographers, and intra- and inter-observer reproducibility analysis, thus, could not be performed. Finally, there were no clinical data of the patients regarding the treatment, follow-up, intra- and post-operative surgical notes, and mortality.

#### Strengths

First, the number of patients included in the study was relatively high. Second, this is the most comprehensive study to investigate the risk factors and CHDs together with echocardiographic findings in this region. Finally, the bias effect was minimized since there was no other tertiary healthcare center where the echocardiographic evaluation was performed and individuals who underwent echocardiography constituted the entire study population.

## Conclusion

In the present study, we found that HHD was the most common comorbidity (39.8%), followed by VHD (34.6%), HFrEF (30.8%), and IHD (24.4%). In addition, the most frequent valvular disease was mitral insufficiency (47.5%) and the most common CHD was ASD (3.2%). Further comprehensive studies with prospective design are warranted to improve inadequate healthcare services and raise public awareness and cultural knowledge in Somalia, a war zone where financial and healthcare opportunities are highly limited.

## Supplementary Information

**Additional file 1.** Demographic characteristics in children and adults patients.

## Data Availability

The datasets used or/and analyzed during the current study are available from the corresponding author on reasonable request.

## Abbreviations

ASD: Atrial septal defect
AV canal defect: Atrioventricular canal defect
CHDs: Congenital heart diseases
CVDs: Cardiovascular diseases
DCMP: Dilated cardiomyopathy
DT: Deceleration time
ECG: Electrocardiography
EF: Ejection fraction
HCMP: Hypertrophic cardiomyopathy
HFrEF: Heart failure with reduced ejection fraction
HHD: Hypertensive heart disease
IHD: Ischemic heart disease
IQR: Interquartile range
LV: Left ventricular
PDA: Patent ductus arteriosus
PS: Pulmonary stenosis
RHD: Rheumatic heart disease
TGA: Transposition of the Great Arteries
ToF: Tetralogy of Fallot
VHD: Valvular heart disease
VSD: Ventricular septal defect
