# Supplementary material for: Spectrum of cardiovascular diseases at a referral tertiary care hospital in Somalia, Mogadishu: an echocardiographic study
Source: BMC Cardiovasc Disord. 2021 Dec 16;21:599. doi: 10.1186/s12872-021-02417-4 (PMC8680378; doi:10.1186/s12872-021-02417-4)
Supplement: Supplementary file 1 — Additional file 1. Demographic characteristics in children and adults patients. [file 12872_2021_2417_MOESM1_ESM.docx]

**Supplementary material.** Demographic characteristics in children and adults patients

|  | All (n:1140 ) | Adults (n:1027 ) | Children (n:113 ) | p-value |
| --- | --- | --- | --- | --- |
| Age, years | 60 (42-70) | 62 (50-71) | 4 (1-10) | **< 0.001** |
| Diabetes mellitus, n (%) | 263 (23.1) | 238 (23.2) | 25 (22.1) | 0.801 |
| Hypertensive heart disease, n (%) | 454 (39.8) | 454 (44.2) | 0 (0) | **< 0.001** |
| Current smoker, n (%) | 57 (5.0) | 52 (5.1) | 5 (4.4) | 0.768 |
| Ischemic heart disease, n (%) | 278 (24.4) | 277 (27.0) | 1 (0.9) | **< 0.001** |
| Heart failure with reduced EF, n (%) | 351 (30.8) | 345 (33.6) | 6 (5.3) | **< 0.001** |
| *Mildly* reduced (41-49%) | 59 (5.2) | 59 (5.7) | 0 (0) |  |
| Reduced (≤40%) | 292 (25.6) | 286 (27.8) | 6 (5.3) |  |
| Valvular heart disease, n (%) | 395 (34.6) | 385 (37.5) | 10 (8.8) | **< 0.001** |
| Mild | 170 (14.9) | 170 (16.6) | 0 (0) |  |
| Moderate | 166 (14.6) | 161 (15.7) | 5 (4.4) |  |
| Severe | 59 (5.2) | 54 (5.3) | 5 (4.4) |  |
| Rheumatic heart disease, n (%) | 84 (7.4) | 71 (6.9) | 13 (11.5) | 0.076 |
| Congenital heart disease, n (%) | 151 (13.2) | 49 (4.8) | 102 (90.3) | **< 0.001** |
| Pulmonary arterial hypertension, n (%) | 260 (22.8) | 236 (23.0) | 24 (21.2) | 0.676 |
| COPD, n (%) | 66 (5.8) | 62 (6.0) | 4 (3.5) | 0.281 |
| Dilated cardiomyopathy, n (%) | 225 (19.7) | 221 (21.5) | 4 (3.5) | **< 0.001** |
| Constrictive pericarditis, n (%) | 9 (0.8) | 8 (0.8) | 1 (0.9) | 0.904 |
| Peripartum cardiomyopathy, n (%) | 21 (1.8) | 21 (2.0) | 0 (0) | 0.125 |
| Tuberculosis, n (%) | 202 (17.7) | 183 (17.8) | 19 (16.8) | 0.791 |

Data are presented as number (n) and percentage (%), or median (interquartile range). p-value was calculated using the Mann-Whitney U-test for continuous variables and the Chi-Square test or the Fisher's exact test for categorical variables as appropriate. p value < 0.05 was considered significant. *Abbreviations*: COPD, Chronic obstructive pulmonary disease; EF, Ejection fraction.
